# Efficient Multi-Sensor Fusion for Cooperative Autonomous Vehicles Leveraging C-ITS Infrastructure and Machine Learning

**DOI:** 10.3390/s25071975

**Published:** 2025-03-21

**Authors:** Jiwon Kwak, Hayoung Jeon, Seokil Song

**Affiliations:** 1 School of Cbersecurity, Korea University, Seoul 02841, Republic of Korea; jwkwak4031@korea.ac.kr; 2 Department of Computer Engineering, Korea National University of Transportation, Daehakro 50, Chungju 27469, Republic of Korea; jeonhy1018@gmail.com

**Keywords:** C-ITS, multi-sensor, fusion, LGBM, machine learning, autonomous driving

## Abstract

The widespread deployment of Cooperative Intelligent Transport Systems (C-ITS) has elevated the need for robust, real-time sensor fusion strategies capable of handling noisy, asynchronous data from multiple infrastructure sensors. In this paper, we propose a two-stage data fusion framework that integrates a grid-based indexing method for efficient duplicate-object detection with a Light Gradient Boosting Machine (LGBM) augmented by an Extended Kalman Filter (EKF). In the first stage, the hybrid EKF–LGBM model mitigates noise, refines object trajectories, and synchronizes sensor streams under varying noise conditions. In the second stage, the grid-based indexing technique rapidly associates objects detected by multiple sensors, merging their measurements into unified state estimates. Extensive experiments—using both synthetic data, where noise scales ranged from 0.5 to 3, and a real-road dataset—confirm that our approach balances near-real-time performance with significantly improved trajectory accuracy. For instance, at a noise scale of 1, the hybrid method outperforms the Unscented Kalman Filter (UKF) while running up to 1.81 times faster, and real-world tests show a 1.54 times RMSE improvement over baseline measurements. By efficiently filtering out noise and minimizing the computational overhead of pairwise comparisons, the proposed system demonstrates practical feasibility with respect to C-ITS applications.

## 1. Introduction

Cooperative Intelligent Transport Systems (C-ITS) provide a wide range of capabilities that enhance traffic efficiency and safety, with sensor data sharing serving as a key component [1,2]. By enabling real-time data exchange among vehicles, infrastructure, and other road users, C-ITS broadens perceptual capabilities and facilitates faster responses to unexpected situations. In particular, the collaborative use of various road infrastructure sensors, such as surveillance cameras and LiDAR, extends the perceptual range of autonomous vehicles. For instance, a method proposed in [1] employs Vehicle-to-Everything (V2X) communication to share real-time data from these infrastructure sensors with autonomous vehicles, thereby improving their object detection range and enabling rapid reactions to unforeseen events.

Despite these benefits, practical deployments of C-ITS still face critical challenges. First, sensor noise often departs from ideal Gaussian assumptions, introducing complex, highly non-Gaussian errors that degrade estimation accuracy. Second, the asynchronous nature of multi-sensor data—where each sensor may operate at different frame rates and latencies—complicates the synchronization of object information. Third, in large-scale deployments, duplicate object identification across multiple sensors becomes computationally expensive, especially when every sensor’s detections must be compared pairwise.

Moreover, C-ITS scenarios feature dynamic and unpredictable traffic conditions, meaning that traditional filtering methods alone fail to capture abrupt maneuvers or nonlinear behaviors effectively. As a result, multi-sensor fusion frameworks must simultaneously address (i) robust noise filtering, (ii) real-time data alignment, and (iii) efficient object association to avoid redundant or conflicting detections.

**Figure 1:**
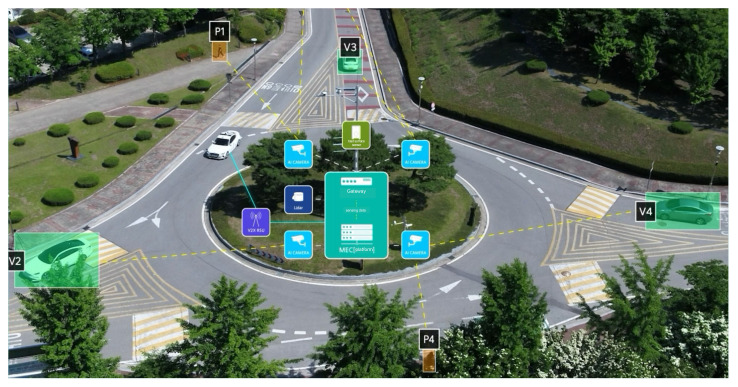
Sensor data-sharing system.

Figure 1 shows a sensor data-sharing system, in which cameras and LiDAR sensors installed along the road transmit object information to a multi-access edge computing (MEC) platform at fixed intervals. The transmitted object information includes the object type, absolute coordinates, speed, direction, and detection confidence. By aggregating these data, C-ITS ensures that autonomous vehicles are consistently updated with real-time environmental information, leading to more accurate and timely decision-making. This comprehensive approach not only benefits individual autonomous vehicles but also contributes to overall traffic management by integrating and distributing sensor data across the transportation network.

Several studies have explored autonomous driving applications in C-ITS. Many focus on using infrastructure sensors for improved road situation awareness via V2X communication. This enables autonomous vehicles to receive real-time sensor data, thereby extending their detection range and reaction time in complex or unpredictable traffic conditions. However, one key challenge lies in identifying whether objects detected by different sensors are the same, which is critical for multi-sensor data fusion. Once this identification is resolved, additional complexities arise from effectively combining the heterogeneous data while accounting for each sensor’s unique characteristics.

Numerous efforts have been devoted to enhancing sensor fusion under noisy and dynamic conditions. For instance, in [3], it is shown that Kalman filter performance varies significantly across different noise distributions, while [4] proposed a two-layer Bayesian fusion protocol that integrates roadside cameras and radar for robust real-time tracking. In [5], a plug-and-play factor graph model for asynchronous data fusion was introduced, outperforming conventional Kalman filter approaches in adaptability and accuracy. Deep learning-based methods have also gained traction. In [6], a residual dense transformer for high-quality multi-sensor image fusion was used; an ensemble learning framework for fault diagnosis was developed in [7]; and in [8], artificial neural networks were employed to address nonlinearities and unknown time-varying errors in autonomous navigation. Collectively, these studies underscore the importance of effective multi-sensor data integration, real-time performance, and noise-aware modeling—key challenges that our work also addresses by focusing on road infrastructure sensors (cameras and LiDARs).

However, these existing methods frequently confront several limitations when deployed at scale in C-ITS, as follows. First, traditional Kalman-based filters often assume noise distributions that do not accurately represent real-world sensor data, especially in urban traffic scenarios with outliers or abrupt changes. Second, associating duplicate objects from multiple sensors can lead to computational bottlenecks if every sensor detection is compared exhaustively. Third, purely data-driven models (e.g., deep neural networks) can capture nonlinearities but may demand large training datasets and high computational resources, posing a challenge for real-time inference on edge devices.

To address these challenges, this paper proposes an integrated multi-sensor data fusion framework that combines a grid-based indexing technique for efficient duplicate detection with a hybrid Extended Kalman Filter (EKF) [9,10] with a Light Gradient Boosting Machine (LGBM) [11] for robust trajectory estimation. Unlike purely theoretical solutions, our framework is designed for real-world operation on an MEC platform, ensuring low-latency performance in large-scale deployments. Specifically, our contributions can be summarized as follows.

First, we introduce an LGBM-augmented EKF that dynamically switches to a machine learning-based correction step whenever the innovation index—representing the discrepancy between predicted and observed measurements—exceeds a threshold. This approach maintains the computational efficiency of EKF while leveraging LGBM’s ability to handle non-Gaussian or highly nonlinear noise. Second, to mitigate the computational overhead of comparing every sensor detection, we use a grid-indexing strategy. Objects are mapped to a grid cell based on their coordinates, and only those in the same or neighboring cells are considered candidates for merging. This significantly reduces redundant comparisons and enables real-time matching, even under high sensor loads. Third, the proposed system has been implemented in an operational environment and validated using both simulated high-density data and real-world traffic datasets. Experimental results confirm near-real-time performance, improved trajectory accuracy, and robust sensor data synchronization under varying noise conditions.

Overall, our proposed EKF + LGBM hybrid method advances state-of-the-art multi-sensor fusion techniques within C-ITS. By addressing key challenges such as object identification across diverse sensors and effective data fusion, this work contributes to safer and more efficient autonomous driving in modern transportation networks. This paper is organized as follows: Section 2 reviews related work in detail, Section 3 presents our proposed fusion method, Section 4 provides experimental evaluations, and Section 5 concludes the paper.

## 2. Related Work

The integration of heterogeneous sensors—including cameras and LiDARs—has been widely explored to enhance estimation accuracy in complex environments. Various methods address the challenges of sensor fusion, noise characteristics, and real-time processing requirements.

A study in [3] examined how different noise distributions affect Kalman filter-based sensor fusion. While Kalman filters are considered optimal under Gaussian noise, uniform noise (often observed in digitized sensor data) induces unique fusion behaviors. The authors underscored the advantages of sensor fusion in reducing RMSE even under difficult noise conditions, offering insights into noise-aware fusion techniques.

A Bayesian-based two-layer fusion protocol for vehicle–road collaborative systems was proposed in [4], integrating roadside cameras and radar. By leveraging historical information to improve real-time tracking and strategically placing roadside sensors to address occlusions, their approach showed robust performance in real-world highway environments—highlighting its potential for large-scale intelligent transportation systems.

In the context of asynchronous multi-sensor scenarios, a plug-and-play factor graph method was introduced in [5]. Their fixed-rate graph model, which incorporates IMU preintegration, facilitates seamless integration of asynchronous data while minimizing computational costs. This method outperforms conventional Kalman filter-based solutions in both accuracy and adaptability under dynamic conditions.

A residual dense transformer (RDT) for multi-sensor image fusion across LWIR, NIR, and VIS spectra was presented in [6]. The proposed model captures both global and local features more effectively than standard CNN methods, leading to substantial improvements in image clarity and fusion quality (particularly relevant for object detection and surveillance applications).

The authors of [7] proposed an ensemble learning-based framework for multi-sensor information fusion in rolling bearing fault diagnosis. Their approach combines a multiscale convolutional neural network (MSCNN) with a fuzzy rank-based decision fusion strategy, achieving high diagnostic accuracy and robustness. By integrating diverse sensor signals, their work emphasizes the growing role of deep learning in industrial fault diagnosis.

An AI-driven approach to multi-sensor integrated autonomous navigation was explored in [8], where artificial neural networks (ANNs) addressed limitations of traditional Kalman filter-based methods, particularly in handling nonlinearities and unknown time-varying errors. This research leveraged multiple heterogeneous sensors—including INS, stellar refraction navigation (SRN), X-ray pulsar navigation (XNAV), and altimeters—and high-precision telemetry data to train a standalone ANN capable of learning complex sensor relationships. Eliminating the need for predefined linear models permitted strong performance under non-Gaussian noise, as well as robust adaptation to evolving conditions.

Research on data fusion methods using the Unscented Kalman Filter (UKF), known to handle nonlinearities effectively, has also been extensively conducted. In [12], the authors proposed a novel multi-sensor hierarchical data fusion algorithm for the attitude observation of wave gliders, utilizing the UKF. Its goal is to enhance the accuracy of attitude estimation by integrating data from various sensors within a hierarchical framework, thereby improving the efficiency of the fusion process.

A multi-sensor fusion underwater localization method that uses the UKF on manifolds was introduced in [13]. This method addresses the challenges of underwater localization by applying the UKF within a manifold framework, accounting for the specific characteristics of the underwater environment. This is particularly useful for underwater robots and underwater exploration equipment.

Additionally, a study in [14] compared the performance of EKFs, UKFs, and particle filters (PFs) in multi-sensor fusion and tracking for autonomous vehicles operating on highways. It evaluates the effectiveness of these filters in accurately estimating the vehicle’s position, velocity, and orientation by fusing data from multiple sensors. The research analyzes the strengths and weaknesses of each filter, providing insights into their suitability for autonomous vehicle applications in complex traffic conditions.

In this paper, our method similarly employs multi-sensor roadside infrastructure sensors for accurate road object detection. Among the several related papers, the study in [4] presents an architecture that aligns closely with ours, making it a particularly apt comparison point. Consequently, we offer a more detailed overview of the two-stage data fusion strategy from [4].

Specifically, ref. [4] begins with a prior representation of each road object’s state, derived from historical sensor readings. It then updates this prior representation with current measurements from one roadside sensor (typically a camera) to generate an intermediate state estimate. In a subsequent step, it applies another update using a second sensor’s data, often from radar, to further refine the object state. This two-stage approach leverages the complementary strengths of different sensor modalities, wherein the camera supplies precise positional and shape details, while the radar provides robust velocity or distance measurements. By iterating these two updates, the authors in [4] not only reduce noise and false positives but also mitigate issues caused by occlusion and multi-path reflections, both of which are common challenges in roadside sensing.

## 3. Proposed Method

To address the challenges, we propose an integrated framework that leverages the Extended Kalman Filter (EKF) and Light Gradient Boosting Machine (LGBM) for robust multi-sensor data fusion. By integrating the strengths of EKF in handling Gaussian noise with the flexibility of LGBM for nonlinear scenarios, our approach mitigates noise, synchronizes multi-sensor outputs, and optimally fuses object information from diverse sensing modalities. A grid-based indexing method also improves efficiency by pruning comparisons to relevant spatial regions, ensuring that multiple sensor detections are accurately matched and merged [15].

In the following subsections, we describe the system architecture, our two-stage fusion method, and the LGBM-augmented EKF (hybrid) in detail. We then introduce our grid indexing approach and demonstrate how these components integrate for comprehensive data fusion. This design not only addresses noise, temporal misalignment, and object association, but also enables real-time performance in dynamic settings such as traffic management and autonomous vehicles.

### 3.1. Overall Architecture and Process of the Two-Stage Fusion Method

**Figure 2:**
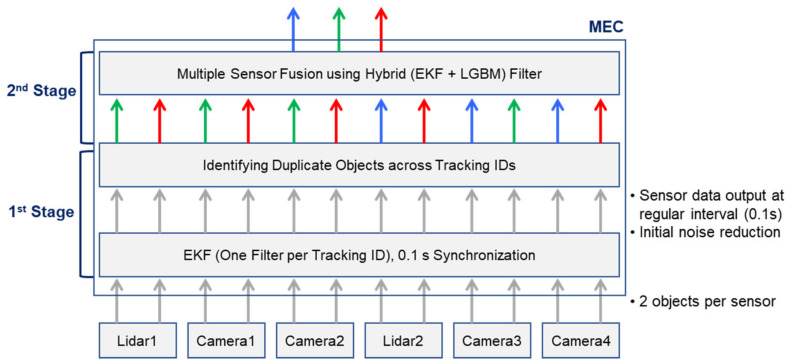
Overall architecture of the proposed method.

The overall architecture of the proposed fusion method is shown in Figure 2. At the bottom, multiple sensors (LiDARs and cameras) transmit object detection data on a frame-by-frame basis at certain intervals. Each detection includes the object’s position, velocity, heading, and type. To maintain consistent timing, all sensors are clock-synchronized, although minor variations in processing or transmission times can still occur. Subsequently, objects identified as identical (indicated by arrows of the same color in the figure) are fused, and the resulting data is then output.

In the first stage, each sensor’s detections are fed into separate EKF instances, where each filter tracks a specific object (identified by a unique Tracking ID). This step refines the per-object estimates using a standard prediction–update cycle. Because multiple sensors can detect the same real-world object independently, the system identifies and merges these duplicate detections into a single unified representation. This merging relies on a grid-based indexing approach to compare objects within the same or neighboring cells, thereby minimizing computational overhead and handling slight localization errors. Each cell is assigned a unique index (e.g., a Hilbert curve [16] or a similar spatial hashing technique), ensuring that overlapping detections can be located efficiently.

In the second stage, the consolidated object tracks are processed by a hybrid fusion filter (EKF + LGBM). Whenever the system detects non-Gaussian noise or nonlinear behavior, it selectively transitions from the EKF’s update step to an LGBM correction, leveraging the machine learning model’s strength in handling outliers or severe nonlinearities. By adapting automatically between EKF-based efficiency and LGBM-driven robustness, this pipeline achieves near-real-time performance under diverse noise conditions. The hybrid filter fuses key object properties—such as position, speed, and direction—into a single, high-precision estimate, even in the presence of conflicting measurements. 

This two-stage design, implemented on the MEC platform, ensures that even if sensor data arrive with slight delays or come from different vantage points, the environment is accurately represented in near real time. As shown in Figure 2, the final output of the hybrid filter (top layer) feeds into downstream applications such as global traffic monitoring, path planning, or other C-ITS services.

### 3.2. LGBM-Augmented Extended Kalman Filter for Robust Trajectory Estimation

This section presents a hybrid trajectory estimation framework that combines the EKF with LGBM. The primary objective is to preserve the EKF’s efficiency under moderately noisy, near-Gaussian conditions while adaptively switching to a machine learning-based correction when measurements deviate substantially from Gaussian assumptions. Table 1 summarizes the key symbols used in the formulation.

We assume *Q* and *R* are either known a priori or can be estimated via sensor calibration. Likewise, *F*, *B*, and *H* are determined from the system’s underlying physical models.

Under normal operating conditions, the EKF performs the following state prediction step:

(1) $$x_{k|k-1}=Fx_{k-1|k-1}+Bu_{k},P_{k|k-1}=FP_{k-1|k-1}F^{T}+Q$$

In Equation (1), $x_{k|k-1}$ is the predicted state at time *k* (before incorporating the latest measurement $z_{k}$), $u_{k}$ is a known control unit (e.g., acceleration or steering), and $P_{k|k-1}$ is the predicted covariance. Here, *Q* accounts for process noise and *F* and *B* come from the system’s state transition model.

Next, we define the innovation $y_{k}$ and its covariance $S_{k}$:

(2) $$y_{k}=z_{k}-Hx_{k|k-1},S_{k}=HP_{k|k-1}H^{T}+R$$

In Equation (2), $y_{k}$ measures the discrepancy between the predicted observation $Hx_{k|k-1}$ and the actual sensor measurement $z_{k}$. The covariance $S_{k}$ reflects the combined uncertainty from both the prediction and measurement processes.

If the system remains close to Gaussian behavior, the standard EKF update is performed:

(3) $$K_{k}=P_{k|k-1}H^{T}S_{k}^{-1},x_{k|k}=x_{k|k-1}+K_{k}y_{k},P_{k|k}=(I-K_{k}H)P_{k|k-1}$$

In Equation (3), $K_{k}$ is the Kalman gain, which balances how much we trust new measurements versus the existing prediction. After this update, the state estimate and its covariance become $x_{k|k}$ and $P_{k|k}$, respectively.

In complex real-world scenarios, highly nonlinear or non-Gaussian behaviors, such as sudden maneuvers or sensor outliers, can cause the EKF to diverge. To address this, the hybrid approach defines a combined score that combines the traditional innovation index and a nonlinearity metric, as follows:

(4) $${score}_{k}=\alpha \left(y_{k}^{T}S_{k}^{-1}y_{k}\right)+\beta n_{k},\;n_{k}=max\left(\frac{\left|\psi_{k}\right|}{\psi_{max}},\;\frac{\left|a_{k}\right|}{a_{max}}\right)$$

In Equation (4), $y_{k}^{T}S_{k}^{-1}y_{k}$ is the innovation index, quantifying how large the measurement residual is relative to its covariance $S_{k}$. Meanwhile, $n_{k}$ is a nonlinearity measure capturing yaw-rate extremes $\psi_{k}$ or rapid acceleration $a_{k}$. The constants α and *β* are tunable weights governing the relative influence of the innovation index versus the system’s nonlinearity.

If ${score}_{k}$ remains below a chosen threshold *T*, the EKF updates normally. In contrast, if ${score}_{k}$ exceeds *T*, we bypass the EKF update to avoid potential filter divergence and instead apply LGBM-based correction. When the score test indicates a severe outlier or strong nonlinearity (${score}_{k}>T)$, state $x_{k|k}$ is replaced with an LGBM output. The LGBM output is generated by a trained LGBM model. The model typically uses current measurement $z_{k}$, predicted state $x_{k|k-1}$, and potentially other features (e.g., road geometry) as inputs. The covariance remains $P_{k|k}=P_{k|k-1}$ in this step, since LGBM does not inherently provide uncertainty estimates.

This hybrid approach enables real-time adaptability. If the innovation index remains below *T*, the EKF step proceeds normally. Otherwise, LGBM correction handles outlier or nonlinear measurements more robustly. By separating these scenarios, we maintain the computational efficiency of the EKF under mild noise while leveraging LGBM’s flexibility in extreme conditions.

**Figure 3:**
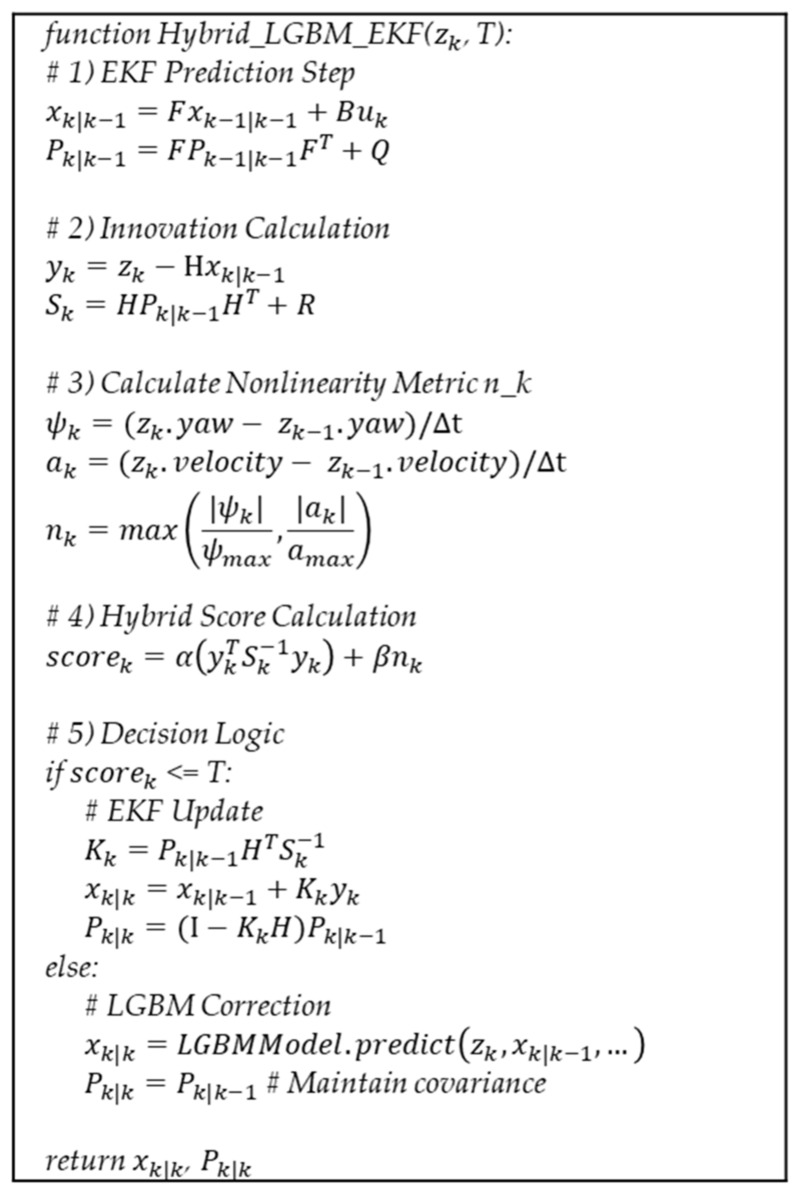
Pseudocode for the hybrid LGBM + EKF filter.

Figure 3 shows the pseudocode for our LGBM-augmented EKF filter. In this pseudocode, at the first step, an EKF prediction step is performed to obtain predicted state $x_{k|k-1}$ and covariance $P_{k|k-1}$. Next, the algorithm calculates innovation $y_{k}$ and $S_{k}$. Here, $y_{k}$ measures the mismatch between predicted observation $x_{k|k-1}$ and sensor reading $z_{k}$, whereas $S_{k}$ quantifies the uncertainty of that mismatch by incorporating both predicted covariance $P_{k|k-1}$ and measurement noise *R*.

A nonlinearity metric, $n_{k}$, captures abrupt yaw or acceleration changes. By comparing the current yaw rate ($\psi_{k}$) and acceleration ($a_{k}$) against their respective maximum values ($\psi_{max}$, $a_{max}$), the method detects whether the vehicle is undergoing highly nonlinear maneuvers. Nonlinearity metric $n_{k}$ is then combined with the standard innovation index to form ${score}_{k}$. As shown in Equation (4), the algorithm sets *α* and *β*, where *α* governs the response to large residuals and *β* adjusts the sensitivity to steep yaw/acceleration swings.

Finally, the decision logic determines whether the system proceeds with a standard EKF update or applies an LGBM correction. If ${score}_{k}$ remains below the threshold *T*, the code assumes that the environment remains near Gaussian or only mildly nonlinear, thereby refining both the state and covariance as in the usual EKF framework. Conversely, if ${score}_{k}$ is greater than *T*, indicating a severe outlier or a highly nonlinear situation, the method bypasses the Kalman gain computation. Instead, it relies on the machine learning-based correction. In this LGBM mode, final estimate $x_{k|k}$ directly replaces the predicted value of the LGBM model, while covariance $P_{k|k}$ remains equal to $P_{k|k-1}$, reflecting the fact that LGBM provides no new covariance information.

The algorithm operates in iterative cycles, alternating between EKF-based updates and LGBM-based corrections. At each time step, the EKF first performs the prediction step to compute $x_{k|k-1}$ and $P_{k|k-1}$. Next, it calculates ${score}_{k}$ from the measurement and predicted state. If this index is below threshold *T*, the EKF executes the standard update. Conversely, if the index exceeds *T*, the LGBM model predicts a corrected state, which then replaces the EKF estimate. Finally, corrected state $x_{k}$ is output as the estimate for the current time step.

To ensure optimal performance, several considerations must be addressed. First, threshold *T* and weights (*α*, *β*) should be determined empirically, based on the standard deviation of the innovation index observed under normal training conditions, thus ensuring accurate switching between the EKF and LGBM. Second, the LGBM model requires careful feature engineering and training on a sufficiently diverse dataset that includes scenarios with significant noise and nonlinearities. The input features should include raw measurements, contextual data, and derived attributes such as velocity and acceleration to maximize predictive accuracy. Finally, computational optimization is critical for real-time applications. Parallel processing should be employed to handle multiple objects simultaneously and reduce latency, and lightweight LGBM models with reduced tree depth and fewer trees should be utilized to balance performance and efficiency.

### 3.3. Grid Indexing for Object Identification

**Figure 4:**
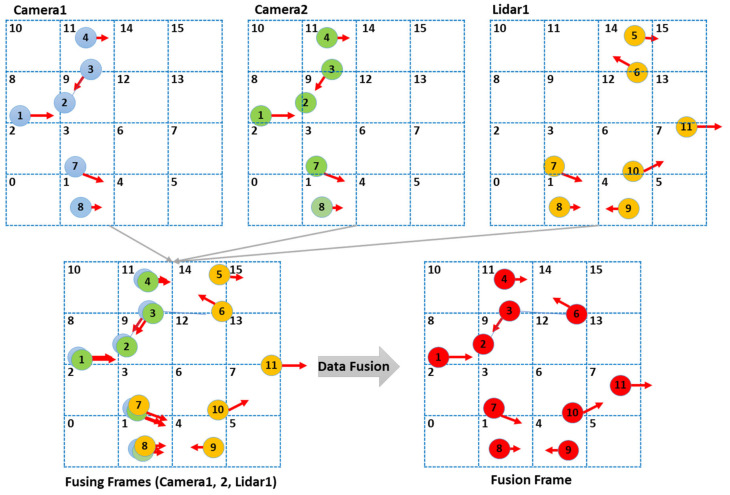
Overall process of identifying duplicate objects and subsequent fusion in a grid index.

The grid-based indexing system provides a computationally efficient framework for object association across multiple sensors. As shown in the revised Figure 4, the detection area is divided into uniform cells, each labeled with a unique numeric index to clarify adjacency. When data arrive from multiple sensors, each detected object is assigned to a cell based on its (*x*, *y*) coordinates. By comparing only those objects residing in the same or neighboring cells, we reduce computational costs while accounting for potential minor misalignments or localization inaccuracies.

In Figure 4, each cell now bears an explicit label (e.g., 0, 1, 2, …), which visually indicates how adjacency is determined. Objects detected by each sensor are represented in different colors. In the fusing frames, overlapping objects with different colors indicate the same object with slight positional errors. Suppose an object lies in Cell 1; the method searches for matching candidates in Cell 1 itself and all immediately adjacent indices (for instance, Cells 0, 2, 3, 6, 4). This localized comparison strategy prevents unnecessary pairwise checks across the entire grid, effectively reducing the algorithm’s complexity from $O(n^{2})$ to approximately O(n) under typical conditions.

**Figure 5:**
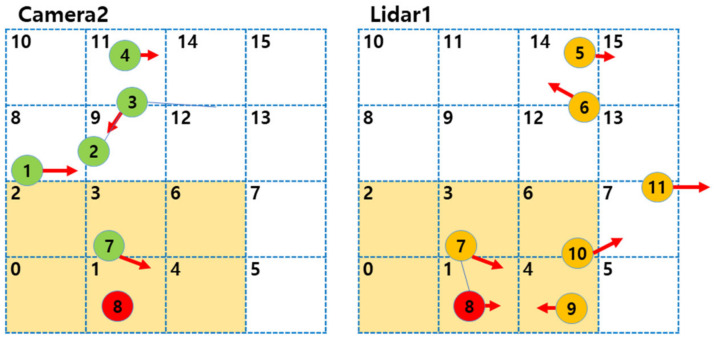
Identifying duplicate objects based on a grid index.

Objects that pass a positional, velocity, or type-based matching criterion are deemed duplicates of the same real-world entity and are merged accordingly. Figure 5 illustrates how the LiDAR-detected “Object 8” in Cell 1 is compared against objects in neighboring cells of Camera 1 and Camera 2. Once their properties are confirmed to match, the corresponding observations are fused into a single state estimate, benefiting from the robust trajectory estimation described in Section 3.2.

This grid-based approach is driven by the assumption that any sensor-level detection error is small enough to ensure that duplicates lie in adjacent or identical cells. If a sensor’s maximum expected localization error is ${err}_{max}$, setting the cell size to at least ${2err}_{max}$ ensures that objects belonging to the same entity cannot be placed in cells beyond immediate neighbors.

By labeling each grid cell explicitly and tying adjacency checks to the cell index structure, we ensure that multi-sensor object association remains both tractable and accurate. This design balances efficiency by eliminating redundant comparisons and robustness by tolerating small localization mismatches, thus facilitating near-real-time fusion in large-scale C-ITS deployments.

### 3.4. Data Fusion

The proposed two-stage fusion process integrates and synchronizes object information from multiple sensors with precision and robustness. As mentioned earlier, in the first stage, the EKF is used to filter noise, refine localization, and synchronize sensor data. In the second stage, the EKF + LGBM hybrid method fuses these partially refined results from different sensors into a unified object representation.

By integrating the EKF and LGBM, the system effectively handles noise, temporal offsets, and data inconsistencies, ensuring that fused object information remains reliable and computationally efficient to compute. Extensive experiments validate its efficacy in real-world conditions, making it a promising approach for traffic management and autonomous navigation applications.

## 4. Performance Evaluation

### 4.1. Experimental Environments and Methods

We conducted a comprehensive performance evaluation encompassing three primary aspects of our proposed approach: sensor data correction and synchronization via the EKF, duplicate detection for effectively identifying the same object across multiple sensors, and a data fusion technique using EKF-LGBM. We designed our experiments to assess both computational efficiency (duplicate detection) and fusion accuracy (EKF-LGBM hybrid method). The first experiment evaluated efficient detection of duplicate objects among multiple sensors, while the second experiment examined data fusion performance across multiple sensors using synthetic and real-world data. Here, a ‘duplicate’ refers to a single real object, e.g., a vehicle, detected by more than one sensor.

All experiments were carried out on an Apple M1 Pro system equipped with 16 GB of RAM, running Python 3.10.x and Apple clang version 16.0.0 (clang-1600.0.26.4) under an arm64-apple-darwin24.1.0 target and a POSIX thread model. This platform provided the computational resources and environment necessary for real-time testing and parallelization. While we do not claim a formal proof of convergence for every noise scenario, we rely on these computational experiments to illustrate the scalability and practical feasibility of our approach, consistent with standard practices in C-ITS research.

### 4.2. Experiments for Detecting Duplicate Objects

In our first experiment, we focused on detecting duplicate objects, instances where two sensors observe the same physical object, at scale. We simulated two sensors capturing 30 frames per second, each detecting up to 500 objects per frame. By varying the number of parallel threads (from 1 to 8), we measured how quickly the proposed grid-based indexing method identifies duplicates compared to a naive $O(n^{2})$ baseline which compares every object pair. To simplify analysis, we assumed complete overlap in the sensors’ detection fields, ensuring that any detected object could be in both. This experiment demonstrates our method’s efficiency gain by restricting comparisons to spatially relevant grid cells and by bypassing repeated checks of previously confirmed duplicates.

The results are shown in Table 2. The results showed that increasing the number of threads from one to eight reduced the proposed method’s duplicate detection time from 0.03 ms to 0.007 ms, thereby demonstrating near-real-time performance even under substantial data loads. By contrast, the naive baseline required 59 ms to compare all possible pairs. The efficiency of the proposed grid-based method arises from significantly reducing the comparison set by focusing only on relevant grid cells, as well as avoiding repeated checks once two objects have been confirmed as duplicates. This advantage is expected to hold even when scaling to 20 sensors on a single MEC platform, indicating that real-time processing is feasible in more extensive deployments.

### 4.3. Data Fusion Experiment Using Synthetic Data and Real Data

In this experiment, we measure the fusion accuracy of the LGBM + EKF hybrid filter. To accomplish this, we evaluate our method on two distinct datasets. The first is a real-world road dataset captured by multiple sensors on a public roadway, in which the mean measurement error is approximately 1.0 m and the standard deviation of measurement error is 1.248 m. The real data provide a practical baseline for assessing the hybrid filter’s performance under everyday urban traffic conditions.

The second dataset consists of synthetic observations (sensor readings) generated via a custom motion model. Unlike fixed-noise real data, synthetic data enable us to systematically vary measurement noise. The synthetic data are generated by adding noise to both the distance and bearing measurements expressed in polar coordinates. Specifically, noise scales of 0.5, 1, 2, and 3 are applied as multipliers to the baseline standard deviations—0.1 m for distance and 0.007 radians for direction. In other words, the noise scale indicates the factor by which the baseline standard deviations are modified. This approach allows us to evaluate the performance of the LGBM + EKF hybrid filter under a variety of conditions, ranging from mild measurement errors at a noise scale of 0.5 to significantly degraded sensor fidelity at a noise scale of 3. Table 3 summarizes the mean and standard deviation of the measurement errors under different noise scales. It is important to note that the mean error and error standard deviation values in Table 2 represent the combined effects of both distance and bearing noise. Therefore, they do not correspond exactly to direct multiples of the noise scale.

By varying the noise scale in the synthetic datasets, we can compare conventional Kalman filter approaches [4] with the EKF + LGBM hybrid in terms of speed and error, demonstrating robustness under non-Gaussian and highly nonlinear scenarios. First, we adjust parameters for the EKF and UKF (process noise covariance *Q*, measurement noise covariance *R*) at each noise scale. We also generate multiple synthetic datasets to determine the best LGBM parameters. For the LGBM + EKF hybrid method, we adjusted *α* and *β* accordingly. The resulting optimal parameters are shown in Table 4.

Table 5 shows the root mean square error (RMSE), mean squared error (MSE), mean absolute error (MAE), and average processing time per update step, demonstrating how each method performs at different noise scales. LGBM achieves the lowest RMSE but suffers from higher latency. Meanwhile, the EKF is very fast but less accurate, and the UKF balances nonlinearity handling at moderate speed. LGBM exhibits the lowest RMSE in all cases, but it is the slowest among the methods tested. Although LGBM is known for relatively fast inference, it may be unsuitable for real-time C-ITS applications due to latency requirements. The EKF runs very quickly but can show an RMSE that is 4–10 times higher than LGBM’s. The UKF handles nonlinear situations better than the EKF but is relatively slower. At small noise scales, the UKF and EKF are similar, but as noise scale increases, the difference becomes more pronounced.

The hybrid approach achieves better RMSE and processing speed than the UKF, depending on the chosen threshold (T in Figure 3). When the noise scale is as low as 0.5 (i.e., minimal noise), the RMSE improves by a factor of 1.1 compared to the UKF, but by a factor of 4.2 relative to the EKF. In terms of processing speed, it is 1.5 times faster than the UKF, yet 0.27 times slower than the EKF. As the noise scale increases to 1.0, the RMSE improves by 1.07 to 1.37 times compared to the UKF, and processing speed increases by 1.16 to 1.81 times. When measured against the EKF with a noise scale of 1.0, the RMSE shows an improvement of 1.9 to 2.44 times, while processing speed is 0.21 to 0.33 times higher.

With a noise scale of 2, the RMSE is 1.04 to 1.18 times better than the UKF, and speed improves by 1.67 to 2.6 times. Compared to the EKF under the same conditions, the RMSE improves by 1.36 to 1.55 times, whereas speed drops to between 0.51 and 0.33 times the EKF’s rate. Even with a substantial increase in noise scale to 3.0, the proposed hybrid method continues to outperform the UKF in both RMSE and processing speed; relative to the EKF, RMSE still improves though processing speed is lower, reflecting a similar trend.

The hybrid method’s performance depends on the threshold parameter, allowing trade-offs between LGBM-level accuracy and EKF-level speed. Overall, even as the noise scale grows from 0.5 to 3, the hybrid approach generally outperforms the UKF in both RMSE and speed while surpassing the EKF in accuracy (albeit at a slower speed). Hence, the method remains viable for C-ITS scenarios where both speed and accuracy are critical.

Even as the noise scale increases substantially from 0.5 to 3.0, the hybrid approach consistently achieves a lower RMSE than both the UKF and EKF. Although its execution speed surpasses that of the UKF, it remains somewhat lower than the EKF’s performance. These findings indicate that the hybrid approach provides sufficiently improved RMSE and speed over the UKF to be practical for C-ITS applications. The hybrid method often strikes a balance between these two extremes, typically outperforming both the EKF and UKF while running faster than a standalone LGBM under many threshold configurations. Moreover, by adjusting the threshold, one can prioritize either accuracy or speed based on specific application requirements.

By analyzing these results, we confirm that the proposed framework remains viable across a broad range of sensor noise conditions. Even as measurement errors rise to nearly ten times the baseline (i.e., at a noise scale of 3), the hybrid approach continues to maintain near-real-time performance while delivering acceptable localization accuracy. This extensive validation reinforces the robustness of our design, demonstrating that neither moderate non-Gaussian noise nor higher-than-anticipated measurement errors lead to catastrophic estimation failures in typical traffic scenarios.

**Figure 6:**
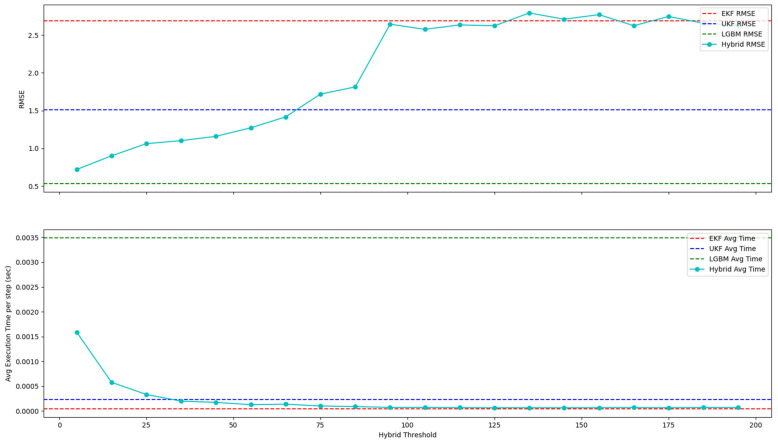
RMSE and average processing time per step according to the threshold of the hybrid approach (at a noise scale of 1).

**Figure 7:**
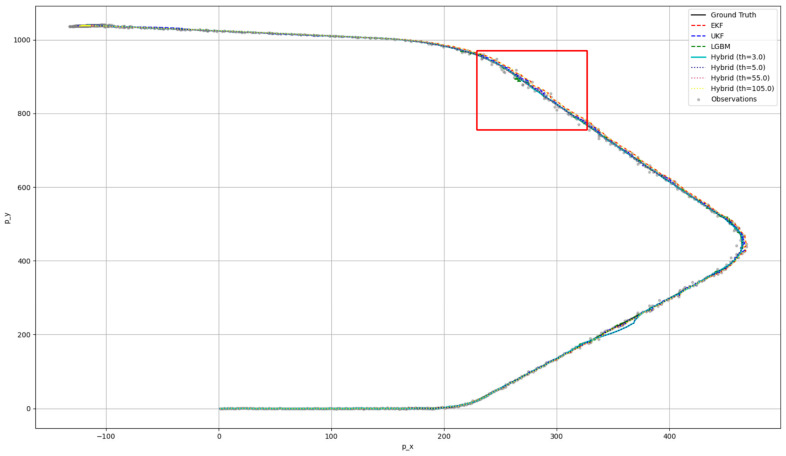
RMSE and average processing time per step according to the threshold of the hybrid approach (at a noise scale of 1; entire synthetic dataset).

**Figure 8:**
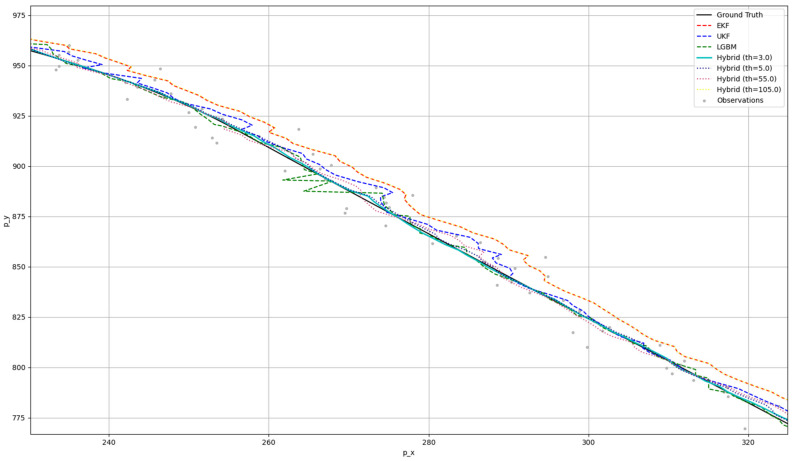
RMSE and average processing time per step according to the threshold of the hybrid approach (at a noise scale of 1; enlarged view of the red-boxed area from Figure 7).

Figure 6, Figure 7 and Figure 8 show how the proposed hybrid filter’s RMSE and processing speed vary with different threshold values at a noise scale of 1. As shown in Figure 6, when the threshold is 65 or below, the resulting RMSE is lower than that of the EKF and the UKF. However, once the threshold exceeds 65, the RMSE converges to that of the EKF. In terms of computation time, using higher thresholds reduces the role of the LGBM, causing the filter’s behavior to resemble that of the EKF. In other words, a lower threshold invokes the LGBM step more frequently (making the filter function more like a standalone LGBM), whereas raising the threshold limits LGBM involvement and shifts the filter toward an EKF-like profile. Consequently, choosing an appropriate threshold is vital for harnessing the strengths of both methods.

Figure 7 visualizes the ground-truth trajectory data, the observed data, and the fusion results produced by the EKF, UKF, LGBM, and hybrid approaches under varying thresholds. Although it depicts the entire trajectory, the differences among these methods are not easily distinguishable. To address this, we magnified the red-boxed region in which errors are relatively large, as shown in Figure 8. The hybrid approach at a threshold of 55.0 delivers the best performance, producing a trajectory that most closely matches the ground truth. Even where the pure LGBM and UKF methods deviate substantially from the true trajectory, the hybrid method set at a threshold of 55.0 maintains the highest accuracy.

In addition to these experiments with synthetic data, testing on our real-world dataset (mean error = 0.985 m, standard deviation = 1.867 m) confirms that the hybrid filter remains effective under practical conditions. These data were obtained through the following procedure. We operated a vehicle equipped with high-precision GPS and an OBU (On-Board Unit) on a road where an infrastructure sensor-based situational awareness system was installed. Communication between the sensor and the MEC uses a custom TCP/IP-based protocol, and the fused data from the MEC are sent to the vehicle from an RSU (Roadside Unit) via a V2X (I2V) protocol. The vehicle then stores the surrounding object information received via the OBU, along with trajectory data collected by the high-precision GPS, in files. The data received through the OBU serve as the observations (sensor readings), and the trajectory collected by the high-precision GPS becomes the ground truth. Table 6 presents the experimental results using real-world data. The RMSE between the collected data and the ground-truth data was 2.107; however, after applying the hybrid filter with a threshold of 20.0, the RMSE improved to 1.364, an enhancement by a factor of approximately 1.54.

Overall, our experiments suggest that the proposed grid-based indexing method reliably identifies duplicates under high data loads and that the hybrid EKF + LGBM effectively corrects noisy measurements, even when the underlying assumptions deviate from ideal Gaussian models. Although we do not provide a formal proof of convergence, the empirical results offer strong support for the system’s scalability and robustness. By unifying these two techniques, we achieve both accurate sensor fusion and low-latency performance, highlighting the system’s potential to handle dense, noisy traffic data in real-world C-ITS applications.

## 5. Conclusions

In this paper, we proposed a machine learning-enhanced multi-sensor data fusion system tailored for real-time traffic monitoring and autonomous driving environments. Our approach integrates grid-based indexing to reduce redundant object comparisons across multiple cameras and LiDAR sensors, complemented by an LGBM-augmented EKF for accurate and robust trajectory estimation under noisy, asynchronous conditions. Experimental results confirm both the scalability and effectiveness of our method. In synthetic datasets, our hybrid approach consistently outperforms the UKF in terms of processing speed and surpasses the EKF in accuracy, even as the noise scale increases significantly (0.5 to 3). For instance, at a noise scale of 1.0, the RMSE improves by 1.07–1.37 times compared to the UKF, while still running 1.16–1.81 times faster. Moreover, testing on real-world traffic data (mean error ≈ 0.98 m) demonstrated a 1.54 times RMSE improvement relative to baseline measurements when using a carefully selected threshold.

These results underscore the hybrid system’s capacity to maintain near-real-time performance and high fusion accuracy under diverse noise distributions and computational loads. By highlighting the practical viability of real-time data processing on an MEC platform, our work contributes to the ongoing advancement of C-ITS research and offers a deployable blueprint for large-scale sensor networks that integrate machine learning components. Future directions include further optimization of grid-cell sizing for diverse road conditions, incorporating additional sensor modalities for broader situational coverage, and exploring deep learning-based approaches to enhance detection and fusion performance.

## Figures and Tables

**Table 1:** Definition of symbols.

Symbols	Definition
$x_{k}\in R^{n}$	State vector at time *k* (e.g., position and velocity).
$u_{k}\in R^{m}$	Control input (e.g., accelerations, wheel commands).
$F\in R^{n\times n}$	State transition matrix.
$B\in R^{n\times m}$	Control–input matrix.
$Q\in R^{n\times n}$	Process noise covariance.
$H\in R^{p\times n}$	Observation model mapping *x_k_* to the measurement space.
$R\in R^{p\times p}$	Measurement noise covariance.
$z_{k}\in R^{p}$	Sensor measurement at time *k*.
$P_{k}\in R^{n\times n}$	Error covariance of the state estimate at time *k*.

**Table 2:** Processing time for duplicate detection.

Category	500 Objects Processing Time (ms)		
	1 Thread	4 Threads	8 Threads
Proposed method	0.03	0.02	0.007
Sequential comparison	59.46	N/A	N/A

**Table 3:** Measurement errors under different noise scales.

Noise Scale	Error Mean (m)	Error Std (m)
0.5	1.779	1.529
1	3.559	3.058
2	7.118	6.116
3	10.676	9.174

**Table 4:** Best parameters per noise scale.

Noise Scale	Best Parameters	
0.5	EKF UKF LGBM Hybrid	q_scale = 2.0, r_scale = 0.1 q_scale = 0.1, r_scale = 0.1 learning_rate = 0.1, max_depth = 7 alpha = 0.5, beta = 1.0
1	EKF UKF LGBM Hybrid	q_scale = 2.0, r_scale = 0.1 q_scale = 0.1, r_scale = 0.5 learning_rate = 0.1, max_depth = 7 alpha = 1.5, beta = 1.0
2	EKF UKF LGBM Hybrid	q_scale = 2.0, r_scale = 0.1 q_scale = 0.1, r_scale = 0.5 learning_rate = 0.1, max_depth = 7 alpha = 1.5, beta = 0.5
3	EKF UKF LGBM Hybrid	q_scale = 2.0, r_scale = 0.1 q_scale = 0.1, r_scale = 1.0 learning_rate = 0.1, max_depth = 7 alpha = 1.5, beta = 1.0

**Table 5:** Performance metrics for each noise scale and approach. For the hybrid approach, the RMSE is smaller than that of the UKF, and the stepwise average time is also lower than that of the UKF, with respect to the threshold (th).

Noise Scale	Approach	RMSE (m)	MSE (m)	MAE (m)	Avg Time per Step (ms)
0.5	ekf	2.541	6.457	1.838	0.00004
	ukf	0.662	0.438	0.38	0.0002
	lgbm	0.59	0.347	0.158	0.004
	hybrid (th = 5.0)	0.602	0.362	0.36	0.0002
1.0	ekf	2.688	7.227	1.857	0.00004
	ukf	1.513	2.29	0.81	0.0002
	lgbm	0.535	0.286	0.148	0.003
	hybrid (th = 35.0)	1.101	1.213	0.682	0.0002
	hybrid (th = 45.0)	1.159	1.342	0.705	0.0002
	hybrid (th = 55.0)	1.272	1.617	0.761	0.0001
	hybrid (th = 65.0)	1.417	2.006	0.838	0.0001
2.0	ekf	3.219	10.363	2.083	0.0001
	ukf	2.455	6.029	1.349	0.0005
	lgbm	0.417	0.174	0.129	0.004
	hybrid (th = 95.0)	2.08	4.33	1.293	0.0003
	hybrid (th = 105.0)	2.119	4.491	1.314	0.0002
	hybrid (th = 115.0)	2.114	4.469	1.321	0.0002
	hybrid (th = 125.0)	2.19	4.795	1.368	0.0002
	hybrid (th = 135.0)	2.245	5.04	1.401	0.0002
	hybrid (th = 145.0)	2.369	5.614	1.476	0.0002
3.0	ekf	4.0	16.003	2.543	0.00005
	ukf	3.873	14.999	2.123	0.0002
	lgbm	0.394	0.156	0.122	0.004
	hybrid (th = 325.0)	3.364	11.32	2.112	0.0002
	hybrid (th = 345.0)	3.384	11.453	2.123	0.0002
	hybrid (th = 365.0)	3.38	11.424	2.119	0.0001
	hybrid (th = 385.0)	3.409	11.623	2.126	0.0001
	hybrid (th = 405.0)	3.467	12.02	2.167	0.0001

**Table 6:** Experimental results using real data (mean error = 0.98478; standard deviation of error = 1.86658).

Approach	RMSE (m)	MSE (m)	MAE (m)
Original Observation Values	2.107	44.397	0.985
Hybrid (th = 20.0)	1.364	18.607	0.52

## Data Availability

The datasets presented in this article are not readily available because the data are part of an ongoing study. Requests to access the datasets should be directed to the corresponding author.
